# Identification of the Scopularide Biosynthetic Gene Cluster in *Scopulariopsis brevicaulis*

**DOI:** 10.3390/md13074331

**Published:** 2015-07-14

**Authors:** Mie Bech Lukassen, Wagma Saei, Teis Esben Sondergaard, Anu Tamminen, Abhishek Kumar, Frank Kempken, Marilyn G. Wiebe, Jens Laurids Sørensen

**Affiliations:** 1 Department of Chemistry and Bioscience, Aalborg University, Fredrik Bajers Vej 7H, DK-9220 Aalborg Ø, Denmark; E-Mails: mbl@bio.aau.dk (M.B.L.); wagmasaei@gmail.com (W.S.); tes@bio.aau.dk (T.E.S.); 2 VTT Technical Research Centre of Finland, P.O. Box 1000, FI-02044 VTT, Finland; E-Mails: Anu.Tamminen@vtt.fi (A.T.); marilyn.wiebe@vtt.fi (M.G.W.); 3 Department of Genetics and Molecular Biology in Botany, Institute of Botany, Christian-Albrechts-University, 24118 Kiel, Germany; E-Mails: abhishek.abhishekkumar@gmail.com (A.K.); fkempken@bot.uni-kiel.de (F.K.); 4 Department of Chemistry and Bioscience, Aalborg University, Niels Bohrs Vej 8, DK-6700 Esbjerg, Denmark

**Keywords:** non-ribosomal peptide synthetases, polyketide synthases, secondary metabolites, transcription factor, *Fusarium*, regulation, marine fungi

## Abstract

Scopularide A is a promising potent anticancer lipopeptide isolated from a marine derived *Scopulariopsis brevicaulis* strain. The compound consists of a reduced carbon chain (3-hydroxy-methyldecanoyl) attached to five amino acids (glycine, l-valine, d-leucine, l-alanine, and l-phenylalanine). Using the newly sequenced *S. brevicaulis* genome we were able to identify the putative biosynthetic gene cluster using genetic information from the structurally related emericellamide A from *Aspergillus nidulans* and W493-B from *Fusarium pseudograminearum*. The scopularide A gene cluster includes a nonribosomal peptide synthetase (*NRPS1*), a polyketide synthase (*PKS2*), a CoA ligase, an acyltransferase, and a transcription factor. Homologous recombination was low in *S. brevicaulis* so the local transcription factor was integrated randomly under a constitutive promoter, which led to a three to four-fold increase in scopularide A production. This indirectly verifies the identity of the proposed biosynthetic gene cluster.

## 1. Introduction

Marine-derived fungi contain a treasure chest of secondary metabolites, of which a considerable number have promising biological or pharmaceutical properties [1,2]. The hunt for discovering novel bioactive secondary metabolites from marine fungi is therefore gaining increased attention. One interesting compound is scopularide A, which was discovered in a *Scopulariopsis brevicaulis* strain isolated from the inner tissue of a marine sponge in Limki Fjord, Croatia [3]. Members of the genus *Scopulariopsis* are generally saprophytes and are common in a number of terrestrial environments, including soil, air, plant debris, paper, and moist indoor environments [4], and have also been isolated in clinical samples [5].

**Figure 1:**
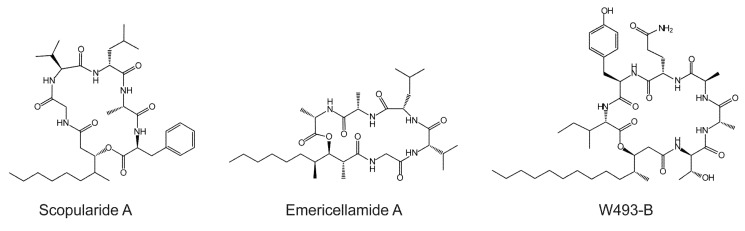
Structure of scopularide A and the related compounds emericellamide A and W493-B.

Scopularide A has specific activity against pancreatic tumor cell lines (Colo357, Panc89) and the colon tumor cell line HT29 [3,6]. It is a cyclic lipopeptide consisting of a carbon chain (3-hydroxy-methyldecanoyl) and five amino acids (glycine, l-valine, d-leucine, l-alanine and l-phenylalanine) [3]. Lipopeptides are cyclic or linear compounds with a fatty acid attached to the *N*-terminal amino acid of the peptide part, which consists of amino acids or hydroxyl acids [7]. The peptide core is synthesized by non-ribosomal peptide synthetases (NRPSs), which consist of modules that facilitate the sequential synthesis of the growing peptide chain. A minimal NRPS module contains an adenylation (A) domain which recognizes the amino acid substrate; a peptide acyl carrier domain (T or PCP) which transfers the amino acid to a condensation (C) domain where the peptide bond is formed [8]. The lipid part, which is attached to the peptide core, can be derived from different sources, including primary lipid metabolism or from polyketide synthases (PKSs) [7]. Like NRPSs, PKSs are multi-domain enzymes in which there are three core domains: the β-ketosynthase (KS), acyltransferase (AT), and acyl-carrier protein (ACP) [9]. The PKSs that provide the carbon chain in lipopeptides contain additional reducing domains such as a dehydratase (DH), enoylreductase (ER), and ketoreductase (KR) and an optional methylation domain (MET). The reduced carbon chain is incorporated into the peptidyl backbone during biosynthesis by a process known as lipoinitiation [10]. Scopularide A is structurally similar to emericellamide A, produced by a marine *Aspergillus* sp. strain [11], and to W493-B, isolated from *Fusarium* sp. [12] (Figure 1). 

Proteins which are involved in biosynthesis of a specific secondary metabolite are often encoded by genes which are located in close proximity forming a cluster [13]. This is also the case for emericellamide A and W493-B, for which the responsible gene clusters have been identified [14,15]. The carbon chains in emericellamide A and W493-B are provided by PKSs, which contain reducing and methylation domains [14,15]. The resulting reduced polyketides are converted to CoA thioesters by acyl-CoA ligases and loaded onto acyltransferases, which shuttles the polyketide intermediates to the first thiolation (T) domain of the NRPSs [14,15]. 

Production of scopularide A by *S. brevicaulis* has been partially optimized in stirred tank bioreactors, resulting in a 29-fold increase in volumetric production compared to non-agitated cultivation [16]. A random mutagenesis approach, based on UV-radiation, has also been applied, which identified one mutant (M26) that had improved scopularide A production characteristics [17,18]. In the present study, the recently sequenced genome of *S. brevicaulis* [19] was used to identify the putative scopularide biosynthetic gene cluster and the local transcription factor has been targeted for overexpression to enhance production of scopularide A.

## 2. Results and Discussion

### 2.1. The Scopularide Gene Cluster Contains a Five Module NRPS and a Reducing PKS

The secondary metabolite gene clusters in *S. brevicaulis* were identified using antiSMASH (antibiotics & Secondary Metabolite Analysis Shell) [20]. The analysis resulted in the identification of three putative multi-modular NRPS genes and thirteen putative mono-modular NRPS genes. One of the three multi-modular NRPS genes (*NRPS2*; g8056), was predicted to contain three adenylation domains and is an ortholog of the siderophore synthetase, *SidC*, responsible for biosynthesis of ferricrocin-like siderophores [21]. The genes encoding the remaining two multi-modular NRPSs contain five (*NRPS1*; g12932) and four (*NRPS3*; g5523) potential adenylation domains [19]. As scopularide A consists of five different amino acids and one carbon chain, the NRPS involved in its biosynthesis would likely contain five adenylation domains. *NRPS3* is an unlikely candidate as it contains only four modules (ATEC-ATC-ATC-ATEC). *NRPS3* is located in a gene cluster that has an ortholog in *Aspergillus niger* (An05g01060), which has not been linked to a product [22]. 

**Figure 2:**
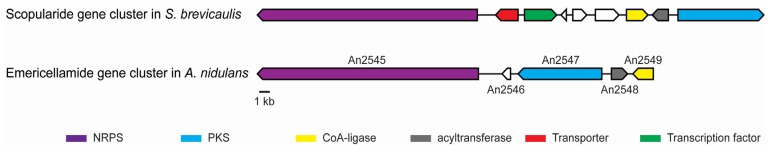
Overview of the scopularide and emericellamide biosynthetic gene clusters in *S. brevicaulis* and *A. nidulans*.

*NRPS1* is the best candidate to encode a synthetase that would synthesize scopularide A in *S. brevicaulis*. Furthermore, *NRPS1* has a similar domain organization as the emericellamide and W493-B synthetases, where the initial module contains a T and C domain for loading and condensation of the reduced carbon chain. The three synthetases also contain an epimerization (E) domain for changing the stereochemistry (l to d stereoisomers) of the first loaded amino acid (threonine in W493-B, glycine in scopularide and emericellamide). The function of this E domain in EasA is unclear since glycine is achiral. An E domain is also found in the third module in *NRPS1*, which correlated with the presence of d-leucine and thereby supports the hypothesis that *NRPS1* is responsible for scopularide biosynthesis. Prediction of the amino acid substrates of the *NRPS*1 adenylation domains by antiSMASH (antibiotics & Secondary Metabolite Analysis Shell) [20] resulted in the consensus prediction ala-nrp-nrp-ala-nrp (nrp = nonribosomal peptide substrate). The PKS/NRPS analyses web-site [23] suggested nrp-asp/asn-glu-pro-nrp, whereas the Non-Ribosomal Peptide Synthase substrate predictor (NRPSsp) [24] predicted val-phe-phe-pro-pro. These unambiguous results are most likely caused by A domains used to build the prediction servers, which primarily are of bacterial origin. Examination of the genes located in the proximity of *NRPS1* identified a putative PKS (*PKS2*; g14542) located 19 kb from *NRPS1* (Figure 2). *PKS2* is predicted to be a reducing PKS, with the same domain organization (KS-AT-DH-MET-ER-KR-ACP) as the emericellamide PKS (EasB; an2547) and sharing 59% identity with the emericellamide PKS. 

The gene clusters in *S. brevicaulis* and *A. nidulans* also both contain a CoA-ligase (g14537) and an acyltransferase (g14538) (Figure 2). The two enzymes are hypothesized to be responsible for activation and loading the reduced polyketide to the NRPS. An additional gene with unknown function is located between the PKS and NRPS in *A. nidulans*, which is not involved in emericellamide biosynthesis [15]. In *S. brevicaulis* five additional genes are located between *PKS*2 and *NRPS1*. These genes are predicted to encode a transporter (g12930), transcription factor (g12928), a copper amide oxidase (g12924), a mannosyl transferase (g12925), and a gene (g12927) with unknown function.

### 2.2. Development of a LC-MS/MS Method for Quantification of Scopularide A

**Figure 3:**
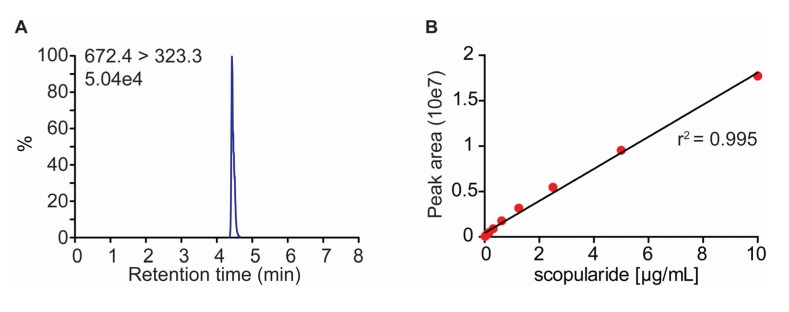
(**A**) Chromatogram of a scopularide A of the lowest concentration of standard (0.02 µg/mL) used in the experiments; (**B**) Standard curve for scopularide A.

Before transforming *S. brevicaulis* a fast method for quantification of scopularide A was developed based on LC-MS/MS (Figure 3). Scopularide could be extracted from *S. brevicaulis* growing on agar medium and examined directly after solids had been removed through centrifugation. The sample run time was 10 min, including calibration, and the quantification was linear over a wide range (0.02–20 µg/mL). Scopularide A ionized very well and the signal-to-noise ratio at 0.02 µg/mL was 90886.

### 2.3. Genetic Manipulation of S. brevicaulis

To verify that the predicted gene cluster is responsible for scopularide biosynthesis we targeted *NRPS1* for deletion using *Agrobacterium tumefaciens* mediated transformation. However, homologous recombination was low and no deletions were observed from the 135 transformants which were analyzed in the study. Rather than screening more transformants for one with a deleted gene we pursued our initial goal, which was to enhance the production of scopularide A. 

Constitutive expression of local transcription factors can be a powerful approach to activate silent gene clusters [25] or to enhance production of secondary metabolites from active gene clusters [26]. This approach has been used in *Fusarium semitectum* for apicidin, which is a lipopeptide produced by a NRPS and a fatty acid synthase [27]. Three mutants, constitutively expressing a transcription factor from the apicidin gene cluster, produced approximately five times as much apicidin as the wild type [27]. To upregulate the scopularide gene cluster, the transcription factor located in the cluster was cloned into a vector, where it was placed under the control of the promoter from the *translation elongation factor 1α* (*TEF*-*1α*) from *A. nidulans*. The expression cassette was introduced into *S. brevicaulis* where it was expected to integrate randomly. Thirty-one transformants were isolated and examined for production of scopularide A by LC-MS/MS in an initial screen (Figure S1). The production of scopularide A was lower in five transformants (<90%), similar in ten (90%–110%) and higher in 16 (>110%). The variation in scopularide production probably reflects variation in the loci of random integration, and possibly of copy number, but was not investigated further since the transformants with improved production were of most interest. Two of the high producing transformants (TF-1-5-1 and TF5-2) were analyzed in submerged culture in bioreactors, under conditions developed for good scopularide A production by LF580 ([16]). 

### 2.4. Production of Scopularide A in Batch Liquid Cultures

**Figure 4:**
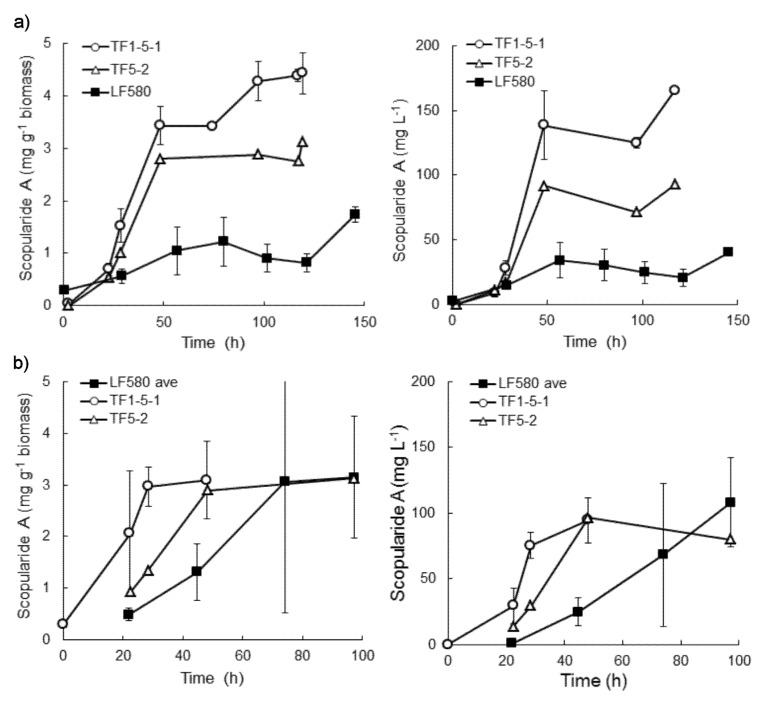
Specific and volumetric scopularide A production by *S. brevicaulis* LF580 and transformants TF1-5-1 and TF5-2, which contain the gene encoding the putative transcription factor for the *NRPS1-PKS2* gene cluster under a constitutive promoter, when grown in (**a**) defined medium and (**b**) complex medium. Error bars represent ± sem (*n* = 2 for TF1-5-1 and *n* = 2 for LF580 in defined medium, *n* = 3 for LF580 in complex medium [16]).

When *S. brevicaulis* LF580, TF5-2 and TF1-5-1 were grown in bioreactors with glucose as the carbon source and (NH_4_)_2_SO_4_ as nitrogen, LF580 produced 1.8 ± 0.1 mg g^−1^ biomass, or 40 ± 2 mg L^−1^ scopularide A in 145 h (Figure 4). In contrast, TF1-5-1 produced 3.4 ± 0.4 mg g^−1^ biomass (138 ± 37 mg L^−1^) within only 48 h (Figure 4), which was significantly more (*p* < 0.10) than produced by LF580 in the same time. TF5-2 produced 2.8 mg g^−1^ biomass (91 mg L^−1^) in 48 h. Both transformants still contained more scopularide A than LF580 after 120 h. Biomass production by the three strains was similar (*p* > 0.10; 23 ± 0.4, 22 ± 0.3 and 23 ± 1 g L^−1^ for LF580, TF1-5-1 and TF5-2, respectively). 

TF1-5-1 produced similar amounts of scopularide A in complex and defined medium and produced significantly more (*p* < 0.10) scopularide A (3.1 ± 0.9 mg g^−1^ biomass, 94 ± 17 mg L^−1^) within the first 48 h than LF580 (1.2 ± 0.5 mg g^−1^ biomass, 25 ± 11 mg L^−1^). However, LF580 continued to produce scopularide A after 48 h, and the final amount produced did not differ from that of TF1-5-1. A similar trend of early production of scopularide A was observed in TF5-2 (Figure 4). The three strains produced similar amounts of biomass in complex medium (*p* > 0.10; 25 ± 1, 26 ± 1 and 24 ± 1 g L^−1^ for LF580, TF1-5-1 and TF5-2, respectively.)

The result indicated that the *NRPS1* gene cluster was probably quite well induced in complex medium, in the conditions described by Tamminen *et al*. 2014 [16], so that addition of the gene encoding the transcription factor under a constitutive promoter resulted only in earlier production of scopularide A. In the defined medium, the cluster did not appear to be as well induced in LF580 as in complex medium and production was considerably lower than in complex medium or in the transformants. 

### 2.5. Model for Biosynthesis of Scopularide A 

**Figure 5:**
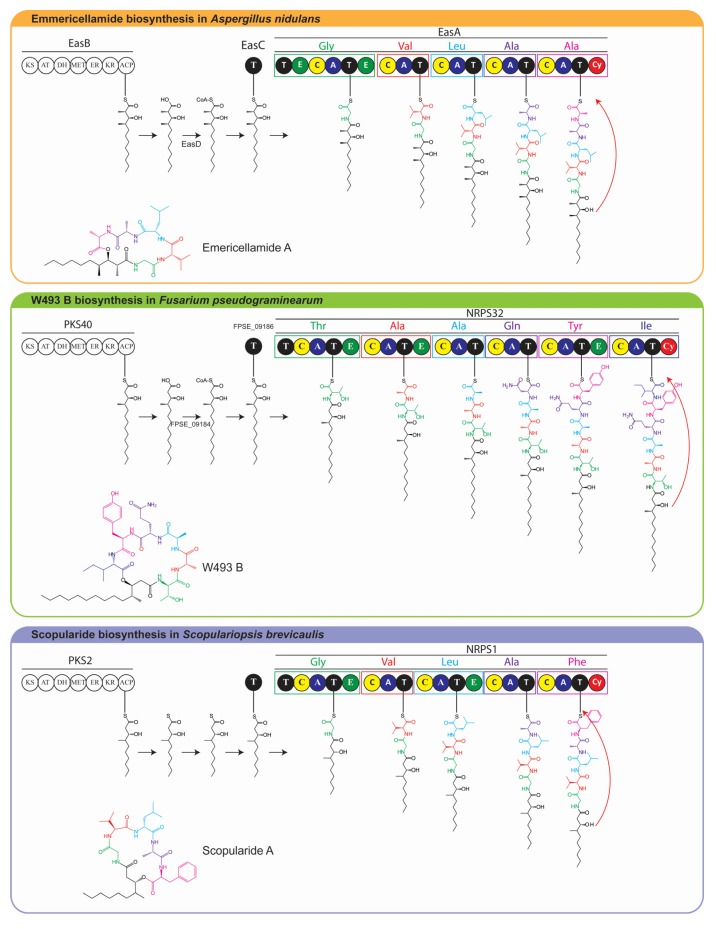
Proposed biosynthetic pathway for scopularide A using emericellamide and W493-B as models [14,15].

The *in silico* analyses of the S*. brevicaulis* genome showed that the most probable biosynthetic gene cluster that could produce scopularide A is *NRPS1*/*PKS2,* although this was not verified by targeted deletion of the biosynthetic genes. It was, however, indirectly proven by the enhanced production of scopularides in the two transformants TF1-5-1 and TF5-2, which contain the gene encoding the putative transcription factor from the gene cluster under a constitutive promoter. Based on the model for emericellamide and W493-B we propose a model for biosynthesis of scopularide A in *S. brevicaulis* (Figure 5), in which the reduced polyketide produced by *PKS2* is loaded to *NRPS1* via the acyl transferase and CoA ligase as described previously [15].

## 3. Experimental Section

### 3.1. In Silico Identification of the Scopularide Biosynthetic Gene Cluster in S. brevicaulis

The genomic and transcriptomic data for *Scopulariopsis brevicaulis* will be available via BioSample accession ID: SAMN03764504 and corresponding BioProject accession ID: PRJNA288424 [19]. Secondary metabolite gene clusters were identified using antiSMASH (antibiotics & Secondary Metabolite Analysis Shell) [20]. The putative scopularide biosynthetic gene cluster was identified *in silico* using the genetic information from the biosynthetic gene cluster of the structurally similar emericellamide from *A. nidulans* [15] and W493-B in *F. pseudograminearum* [14].

### 3.2. Strains and Genetic Manipulation of the Scopularide Biosynthetic Gene Cluster

*Scopulariopsis brevicaulis* LF580 was obtained from A. Labes, from the culture collection of the Kiel Center for marine natural products at GEOMAR, Helmholtz Centre for Ocean Research Kiel. Stock cultures were maintained as conidia suspended in 20% v/v glycerol, 0.8% w/v NaCl with ~0.025% v/v Tween 20 at −80 °C.

**Figure 6:**
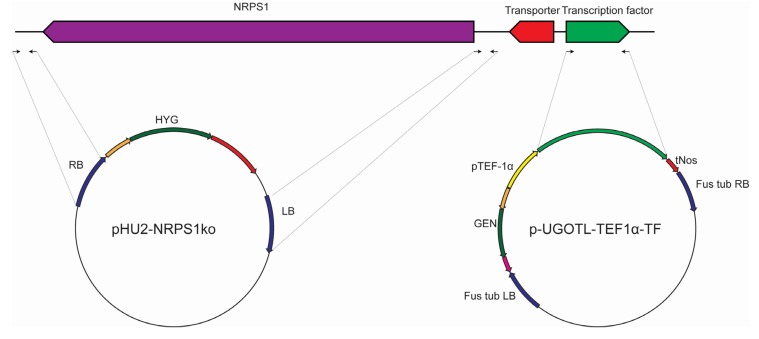
Generation of vectors for targeted deletion of *NRPS1* and for overexpression of the cluster transcription factor. The deletion vector pHU2-*NRPS1*KO was generated by cloning 707–717 bp from the border regions of *NRPS1* into a vector carrying a hygromycin resistance cassette [28]. To generate the transcription factor expression vector, the gene was cloned into a vector carrying a geneticin (G418) resistance cassette [29] and a promoter from the *A. nidulans* translation elongation factor 1α (pTEF-1α). This vector also contained border sites from the *F. graminearum* β-tubulin [29]. Both vectors were introduced into *S. brevicaulis* by *Agrobacterium tumefaciens* mediated transformation.

To verify that *NRPS1* is responsible for biosynthesis of scopularides, two approaches, illustrated in Figure 6, were considered. For the first approach, a knock out cassette was constructed in which the border regions of *NRPS1* (707 and 717 bp) were amplified by PCR using primers *NRPS1*-KO1–KO4 (Table 1) and cloned into a knock out cassette with hygromycin resistance by USER Friendly cloning [28] (Figure 6). 

To overexpress the putative transcription factor in the scopularide biosynthetic gene cluster the gene was amplified by PCR using primers TF1 and TF2 (Table 1). The transcription factor was cloned into an expression vector, in which it was controlled by the constitutive promotor from the *A. nidulans* translation elongation factor 1α (TEF-1α), and which contained a geneticin (G418) resistance cassette. The vector was derived from mutation studies in *Fusarium* and contained border sites for integration at the β-tubulin locus [29]. The border sites are however insignificant for the present study as *S. brevicaulis* has a very low level of homologous recombination. 

**Table 1:** Primers used to generate vectors.

Primers	Sequence ^a^	Amplicon
*NRPS1*-KO1	5′- **TAAACGGCGCGCCGG**CGATAACCCGGTCCGACCATAC	707 bp
*NRPS1*-KO2	5′- **AGTGCGCGATCGCGG**GTAGGTGCCCGTTTGTCGTTTCAAATG	
*NRPS1*-KO3	5′- **ATTAAACCCACAGCG**GGTTGCTCTGGTGGGGCCGAGGGT	717 bp
*NRPS1*-KO4	5′- **GTGAATTCGAGCTCG**CGTGGTGTTCCACACCAAGATTGGG	
TF1	5′-GC **GGATCC**ATGACAAGCTGGCAATTG	2288 bp
TF2	5′-GC **GTCGAC**CTACCCAAAAAGAGATCG	

^a^ USER Friendly cloning sites and restriction enzyme digestion sites in bold.

*S. brevicaulis* LF580 was transformed by *Agrobacterium tumefaciens* mediated transformation using a protocol derived from *Fusarium* [29,30], with the only modification being that the transformants were recovered on Czapek dox medium with yeast extract (CYA) [4] instead of *Fusarium* defined medium (DFM). This change in medium was necessary as *S. brevicaulis* grew poorly on DFM and we did not obtain any transformants.

### 3.3. Solid State Growth of S. brevicaulis Transformants and Extraction of Scopularides 

To screen transformants for scopularide production they were cultivated on CYA in 6.5 mm diameter Petri dishes for 10 days at 25 °C in the dark. Six plugs of agar and mycelium (5 mm) were excised and transferred to 10 mL glass tubes, in which they were extracted in 1 mL methanol by ultrasonication for 45 min. Mycelium and agar debris were removed from the extracts by centrifugation in micro centrifuge tubes for 1.5 min at 12,000 rpm. The extracts were then transferred to HPLC vials and analyzed by LC-MS/MS.

### 3.4. Quantification of Scopularide A

In order to quantify scopularide production in the *S. brevicaulis* transformants a LC-MS/MS method was developed using a dionex UltiMate 3000 UHPLC system (Thermo Fisher Scientific, Idstein, Germany) connected to a Thermo Vantage triple stage quadrupole mass spectrometer (Thermo Fisher Scientific, San José, CA, USA) with a heated electrospray ionization probe. The calibration for scopularide A was performed using a pure standard, kindly provided by Dr. Antje Labes (GEOMAR). The settings for selected reaction monitoring (SRM) transitions for scopularide A were automatically optimized using a 10 µg/mL solution (Table 2). 

**Table 2:** Parameters for selected reaction monitoring (SRM) transitions for scopularide A.

	RT ^a^	Precursor Ion	Product Ions ^b^	S-Lens	CE ^c^
Scopularide A	4.45	672.4 [M + H]^+^	323.3/436.4	121	28/21

^a^ Retention time; ^b^ Quantifier/qualifier/qualifier ions; ^c^ Collission energy for product ions.

The LC was performed with a kinetex phenyl-hexyl column (2.6 μm, 2-mm i.d. × 100-mm, Phenomenex, Torrance, CA, USA) using a constant flow of a 0.4 mL/min and gradient system consisting of A (H_2_O:acetic acid; 99:1) and B (MeCN:H_2_O:acetic acid; 89:10:1), both buffered with 5 mM ammonium acetate. The gradient started at 40% B increasing to 100% over 6.5 min, which was maintained for one minute before reverting to 40% B acetonitrile in one minute and recalibrated for 1.5 min. The following ion source parameters were used for detection of scopularide A: spray voltage (4.5 kV), vaporizer temperature (350 °C), nitrogen sheath gas pressure (14 arbitrary units), nitrogen auxiliary gas pressure (9 arbitrary units), and capillary temperature (270 °C). Argon was used as the collision gas and set to 1.5 mTorr. Alternatively, scopularide A was quantified by UPLC with UV detection as described previously [16].

### 3.5. Cultivation of S. brevicaulis LF580, TF5-2 and TF1-5-1 in Bioreactors

*S. brevicaulis* LF580 and transformants TF5-2 and TF1-5-1 were grown in Biostat Qplus bioreactors (1 L max. working volume, Sartorius AG, Göttingen, Germany), as described by Tamminen *et al*. [16]. The defined medium described by Vogel [31] was modified, to provide 40 g L^−1^ glucose as carbon source, 8.3 g L^−1^ (NH_4_)_2_SO_4_ as nitrogen, and 30 g L^−1^ Tropic Marin^®^ Sea Salt. Alternatively, complex medium contained 3 g L^−1^ yeast extract, 3 g L^−1^ malt extract, 5 g L^−1^ soy peptone and 30 g L^−1^ Tropic Marin^®^. Pre-cultures (10% of the reactor volume) were grown in flasks in the same medium as was used in the bioreactor, supplemented with 4 g L^−1^ agar to obtain filamentous growth. Biomass was measured after centrifugation, washing in water, and freeze-drying. 

When *S. brevicaulis* was grown in bioreactors the freeze-dried mycelium was extracted using ethyl acetate, as described by [16].

## 4. Conclusions 

Using the genome sequence of *S. brevicaulis* we predicted the putative scopularide biosynthetic gene cluster, which includes gene encoding an NRPS, a PKS a CoA-ligase and an acyltransferase. Targeted deletion of *NRPS1* was not achieved, but we indirectly verified that this gene cluster was responsible for scopularide biosynthesis by constitutively expressing a transcription factor located in the gene cluster. This led to a three to four-fold increase in scopularide A production (Figure 4) in at least two transformants. The relatively low level of increase is similar to that observed previously with constitutive expression of transcription factors from lipopeptide biosynthetic gene clusters. The results indicate that the scopularide gene cluster was relatively well expressed in the parental strain during growth in complex medium and that additional transcription factor was not needed for production in complex medium. Overexpression of the transcription factor would, however, be useful for production in chemically defined medium.

## Supplementary Files

Supplementary File 1
