# Supplementary material for: Identification of the Scopularide Biosynthetic Gene Cluster in Scopulariopsis brevicaulis
Source: Mar Drugs. 2015 Jul 14;13(7):4331–43. doi: 10.3390/md13074331 (PMC4515620; doi:10.3390/md13074331)
Supplement: Supplementary File 1 [file marinedrugs-13-04331-s001.docx]

**Supplementary Information**

**Figure S1.** Screen of scopularide A production in colonies of *S. brevicaulis* wild
type (control, red) and 31 strains transformed with an expression cassette carrying the
local transcription factor controlled by the *A. nidulans* TEF-1α promoter. Two strains (marked with stars) were assessed in bioreactors.

© 2015 by the authors; licensee MDPI, Basel, Switzerland. This article is an open access article distributed under the terms and conditions of the Creative Commons Attribution license (http://creativecommons.org/licenses/by/4.0/).
